# Harmful to Parents, Harmless to Offspring: Lethal and Transgenerational Effects of Botanical and Synthetic Insecticides on the Egg Parasitoid *Trichogramma atopovirilia*

**DOI:** 10.3390/insects16050493

**Published:** 2025-05-05

**Authors:** Emile Dayara Rabelo Santana, Leonardo Vinicius Thiesen, Leandro do Prado Ribeiro, Tamara Akemi Takahashi, José Roberto Postali Parra, Pedro Takao Yamamoto

**Affiliations:** 1 Department of Entomology and Acarology, “Luiz de Queiroz” College of Agriculture, University of São Paulo (ESALQ/USP), Piracicaba 13418-900, São Paulo, Brazil; leonardo.thiesen@usp.br (L.V.T.); tamaratakahashi@gmail.com (T.A.T.); pedro.yamamoto@usp.br (P.T.Y.); 2 Research Center for Family Agriculture, Agricultural Research and Rural Extension Company of Santa Catarina (CEPAF/EPAGRI), Chapecó 89801-970, Santa Catarina, Brazil; leandroribeiro@epagri.sc.gov.br

**Keywords:** acetogenins, limonoids, biological control, integrated pest management, selectivity

## Abstract

This study investigated the lethal and transgenerational effects of botanical and synthetic insecticides on the egg parasitoid *Trichogramma atopovirilia*, an important natural enemy of *Spodoptera frugiperda* in Brazil and beyond. The treatments were assessed for their impact on parasitism, emergence, sex ratio, and flight capacity of adults exposed to contaminated eggs. The botanical insecticide ESAM (ethanolic seed extract of *Annona mucosa*) significantly reduced the parasitism in the F_0_ generation by 99.76%, categorizing it as toxic. Anosom^®^ [acetogenins (annonin as a major component)] and Azamax^®^ [limonoids (azadirachtin + 3-tigloilazadirachtol)] also caused substantial reductions (99.13% and 92.36%, respectively) in the parasitism rate. EFAMON (ethanolic leaf extract of *Annona montana*) reduced the parasitism by 62%, while the synthetic insecticide Premio^®^ (chlorantraniliprole) resulted in a 28.21% reduction. In the F_1_ generation, emergence rates for EFAMON, Azamax^®^, and Premio^®^ exceeded 70%, showing no significant differences from the negative control (82%), while Anosom^®^ resulted in a lower emergence rate of 61.39%. No significant effects were observed on sex ratio or parasitism in the F_1_ and F_2_ generations. Most adults reached high flight capacity (above 80%). These results indicate that while ESAM was toxic, the other treatments showed no transgenerational effects. Our findings contribute to understanding insecticide selectivity and highlight the importance of such studies for the sustainable management of *S. frugiperda* within integrated pest management programs.

## Simple Summary

*Trichogramma atopovirilia* is an egg parasitoid used for the biological control of fall armyworm (*Spodoptera frugiperda*), a major agricultural pest. To better understand how different insecticides affect this natural enemy, we evaluated the lethal and transgenerational effects of botanical and synthetic insecticides. The ethanolic seed extract of *Annona mucosa* (ESAM) was highly toxic, almost completely reducing parasitism in the exposed generation. Other botanical insecticides also reduced parasitism but had no significant impacts on subsequent generations. The tested synthetic insecticide had a moderate effect on the exposed generation without relevant transgenerational effects. Overall, while some botanical insecticides affected *T. atopovirilia*, most did not compromise its long-term biological control potential. These findings underscore the importance of insecticide selectivity studies in ensuring sustainable integrated pest management strategies.

## 1. Introduction

The fall armyworm, *Spodoptera frugiperda* (J.E. Smith) (Lepidoptera: Noctuidae), is one of the most significant agricultural pests due to its ability to adapt to different environments and host plants. This characteristic allows it to cause significant damage to economically important crops, especially corn, one of its preferred hosts, with yield losses reaching up to 94.8% [1,2]. The rapid evolution of resistance to insecticides and *Bt* corn intensifies the challenges in its management [3]. In this context, integrated pest management (IPM) programs emphasize the association of chemical and biological control methods as an alternative to mitigate the impact caused by this pest [4,5].

Egg parasitoids of the genus *Trichogramma* (Hymenoptera: Trichogrammatidae) are important tools in biological control [6,7]. Among the species of this genus, *Trichogramma atopovirilia* Oatman & Platner stands out for its wide distribution and ability to parasitize eggs of different lepidopteran pests, including *S. frugiperda* [8,9,10,11]. Jalareño-Teniente et al. [9] demonstrated that *T. atopovirilia* achieves high parasitism rates under laboratory conditions. Compared to *Trichogramma pretiosum* Riley, another parasitoid commonly used for *S. frugiperda* management, *T. atopovirilia* exhibits superior performance, particularly in overcoming the physical barrier formed by the scales covering the eggs [12].

Synthetic insecticides represent another important tool for controlling *S. frugiperda*. However, previously reported cases of resistant populations to the main active ingredients available, along with concerns about environmental impacts associated with this method, have driven the search for alternatives, including the adoption of botanical insecticides [4,13]. These natural compounds are often described as less toxic to non-target organisms and less harmful to the environment [14]. Plants from the Annonaceae family, including *Annona mucosa* Jacq., *Annona montana* Macfad., and *Annona squamosa* L., have demonstrated insecticidal activity against *S. frugiperda* [15,16,17]. The toxicity of these botanical compounds is primarily attributed to acetogenins, a class of bioactive molecules with diverse modes of action, including cytotoxic effects, which cause changes in midgut epithelium and digestive cells of insects [18,19].

The integration of biological control using *T. atopovirilia* and chemical control with botanical insecticides is a promising strategy for managing *S. frugiperda* [5,6]. However, *T. atopovirilia* can be susceptible to insecticide exposure, whether through direct contact or residual effects, which may negatively impact its population and reduce its effectiveness [20,21]. Therefore, it is important to conduct studies that evaluate the selectivity of botanical insecticides to ensure compatibility between these two control methods and promote balance in agroecosystems [22,23,24,25,26,27].

Selective insecticides are those that are more toxic to pests than to beneficial organisms [28]. The selectivity of these insecticides is evaluated through ecotoxicological studies, often following protocols established by the International Organization for Biological Control (IOBC). These protocols include tests that assess both the lethal and sublethal effects of the insecticides [29,30,31,32]. Additionally, it is important to consider transgenerational effects, as the impacts of an insecticide can be transmitted to subsequent generations, even if the previous generation did not suffer direct effects [33,34]. While previous studies have investigated the effects of synthetic insecticides on *T. atopovirilia*, this is the first approach involving botanical insecticides and their impacts across generations [22,25,26,27].

In light of this context, this study aimed to assess the compatibility between botanical and a synthetic insecticide with the egg parasitoid *T. atopovirilia*. We hypothesize that botanical insecticides from the Annonaceae family may exhibit lower toxicity to *T. atopovirilia* compared to the synthetic insecticide. To test this hypothesis, we assessed the lethal effects of botanical insecticides and the synthetic insecticide on *T. atopovirilia*, sublethal impacts on parasitism, emergence, sex ratio, and flight capacity, as well as transgenerational effects by analyzing offspring performance. Understanding these effects can contribute to the integration of pest management tactics in the framework of *S. frugiperda* management, which is aligned with the principles of IPM, promoting agricultural sustainability and food safety.

## 2. Materials and Methods

### 2.1. Maintenance of Trichogramma atopovirilia Colony

*Trichogramma atopovirilia* were obtained from a laboratory colony maintained by the Insect Biology Laboratory at the University of São Paulo, Luiz de Queiroz College of Agriculture (Piracicaba, São Paulo, Brazil). The adults were kept in 50 mL glass tubes sealed with plastic film (Tecfilm, Bom Retiro, SP, Brazil) and provided with droplets of pure bee honey as a food source. For colony maintenance, UV-sterilized eggs of the alternative host *Ephestia kuehniella* Zeller, 1879 (Lepidoptera: Pyralidae), supplied by Koppert Biological Systems (Charqueada, SP, Brazil), were used. The eggs were affixed to 7.6 × 12 cm cardboard sheets using double-sided adhesive tape (Adere, Sumaré, SP, Brazil) and offered to the adult *T. atopovirilia* for 48 h to allow for parasitism. Subsequently, the parasitized sheets were transferred to new glass tubes where they remained until adult emergence. At that point, the entire process was repeated. The rearing was maintained in climate chambers (Eletrolab, São Paulo, SP, Brazil) under controlled conditions of temperature (25 ± 1 °C), relative humidity (60 ± 10%), and 14 h photophase.

### 2.2. Treatments and Concentrations

The insecticides selected for assessing selectivity on *T. atopovirilia* exhibit insecticidal activity against *S. frugiperda* in previous studies [15,16,17,35]. Five insecticides were selected and classified into three categories. The first group included two non-commercial pre-formulations: one based on the ethanolic seed extract of *Annona mucosa* (ESAM) and another composed of the methanolic fraction of the ethanolic leaf extract from *Annona montana* (EFAMON). The second group consisted of two commercial botanical insecticides: Anosom^®^ 1 EC, which is based on acetogenins (annonin as a major active ingredient), and Azamax^®^ 1.2 EC, based on azadirachtin and 3-tigloylazadirachtol. The third group included the commercial synthetic insecticide Premio^®^ SC, which has chlorantraniliprole as its active ingredient. The control treatment consisted of distilled water and the solvents used in the pre-formulations: Triton^®^ X-100 (0.1%, *v v*^−1^), Tween^®^ 80 (1%, *v v*^−1^), acetone + methanol [1% (*v v*^−1^), in a 1:1 ratio] (Êxodo Científica Química Fina Indústria e Comércio Ltd., Sumaré, SP, Brazil). 

All bioassays were conducted using the lethal concentration that causes 90% mortality (LC_90_) in *S. frugiperda*. The procedure for determining the LC_90_ for each treatment is described in Supplementary Material S1, while the corresponding values and additional information are presented in Table 1.

### 2.3. Bioassays

**Figure 1:**
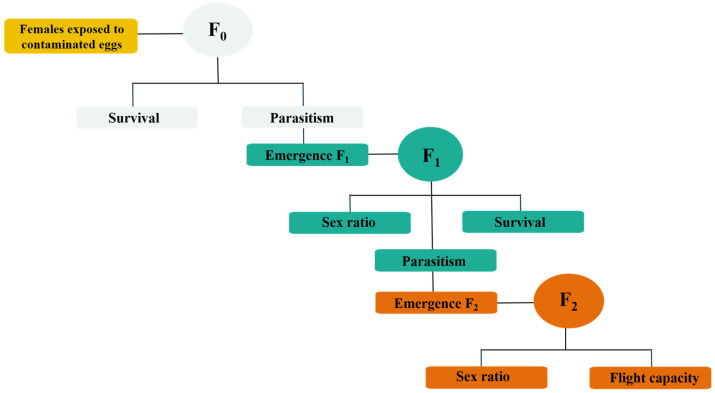
Diagram of selectivity bioassays on *T. atopovirilia*.

Selectivity bioassays were performed to assess the lethal and transgenerational effects of insecticides on *T. atopovirilia* through contact exposure with contaminated eggs (Figure 1). All experiments were conducted under controlled conditions (temperature: 25 ± 1 °C, relative humidity: 60 ± 10% and a photophase of 14 h) following a completely randomized design.

#### 2.3.1. Survival and Parasitism of the F_0_ Generation (Parental)

To assess the effects of the treatments on the survival of *T. atopovirilia*, cards containing approximately 150 UV-sterilized eggs of the factitious host (*E. kuehniella*) were immersed for 10 s in solutions with the LC_90_ of the treatments. After drying, the cards were placed in 25 mL glass tubes, and a 24 h old female was introduced to ensure contact with the contaminated eggs for 24 h. Following this period, the contaminated cards were replaced daily with untreated cards, and the survival of the parasitoid was monitored until the death of the last individual. Pure honey was provided as food for the parasitoid. To assess the parasitism capacity of the females exposed to the treatments, the cards containing eggs collected daily from the survival bioassay were kept in 25 mL test tubes until the eggs darkened, indicating parasitism. The number of parasitized eggs (darkened eggs) was then counted using a stereoscopic microscope (Stereo-Zoom SZ/SZT series, BEL Engineering, Newcastle upon Tyne, UK). The experiment included 30 replicates per treatment, with one female per replicate and 150 eggs were offered daily to each female.

#### 2.3.2. Transgenerational Effects on F_1_ and F_2_ Generations

The eggs parasitized by females exposed to treatments were kept until the emergence of F_1_, with evaluations of the emergence rate and sex ratio. The emergence rate was calculated based on the number of eggs with exit holes in relation to the total number of darkened eggs, while the sex ratio was assessed by differentiating antennae [36]. F_1_ females were individually placed in test tubes (25 mL) and received daily cards containing eggs of the factitious host (*E. kuehniella*) for assessing parasitism. Their survival was recorded until the last female died. Following the same procedures described previously, the emergence rate and sex ratio of the F_2_ generation were also assessed. The transgenerational effects experiments were conducted with 15 replicates per treatment.

#### 2.3.3. Flight Capacity of the F_2_ Generation

To investigate the effects of insecticides on the flight capacity of the F_2_ generation, females from the previous generation were individually placed in test tubes (25 mL) and exposed to cards containing uncontaminated *E. kuehniella* eggs for parasitism. Close to the parasitoid emergence date (two days before), these cards were placed in test tubes (50 mL) positioned in the center of flight cages [37,38].

The cages consisted of plastic tubes (18 cm in height × 11 cm in diameter) internally lined with black cardstock and sealed at the base with an acrylic lid covered with black adhesive paper. A non-toxic entomological glue ring (0.5 cm wide) was positioned inside the cage, 3.5 cm from the base. Additionally, the upper transparent acrylic lid was coated internally with entomological glue.

Three days after the estimated emergence date, the number of insects that reached the top of the cage (flyers), those trapped in the glue ring (walkers), and those remaining at the base (non-flyers) were recorded. The experiment was conducted using a completely randomized design, with six replicates per treatment. Each replicate contained approximately 50 parasitized eggs. The percentage of adults in each section of the cage was calculated based on the total number of insects found.

### 2.4. Statistical Analysis

Survival data were analyzed using survival curves with the ‘*survival*’ package [39] in R 4.2.1 [40]. The treatment effects were assessed using the Cox proportional hazards regression model fitted to estimate the lethal time necessary to kill 50% of the individuals (LT_50_). Differences in survival among treatments were compared using the log-rank test at a 5% significance level [41]. For parasitism data, generalized linear models (GLMs) were fitted to compare treatments after 24 h of exposure and the cumulative data at the end of the experiment (16 days). The same approach was used to compare the flight capacity of *T. atopovirilia,* using a two-factor GLM (treatments × location). Models assuming binomial distribution were used, with corrections for overdispersion when necessary, including quasi-binomial and beta-binomial models. The goodness of fit of the models was evaluated using simulated envelopes within a 95% confidence interval, assessed through half-normal plots generated by the *‘hnp’* package [42]. Treatment comparisons were performed using deviance analysis, and when significant differences were detected, means were compared using Tukey’s test at a 5% significance level.

The treatments were classified into toxicological categories based on their reductions in the beneficial capacity of *T. atopovirilia*, considering both parasitism and adult emergence, following the IOBC/WPRS recommendations. The classifications were defined into four categories: class 1 = innocuous (reduction < 30%); class 2 = slightly toxic (30% to 80% reduction); class 3 = moderately toxic (reduction > 80% to 99%); and class 4 = toxic (reduction > 99%). The reduction percentage (R) was calculated using the formula: R = 100 − (insecticide treatment value × 100 ÷ control value) [32].

## 3. Results

### 3.1. Survival and Parasitism of the F_0_ Generation (Parental)

**Figure 2:**
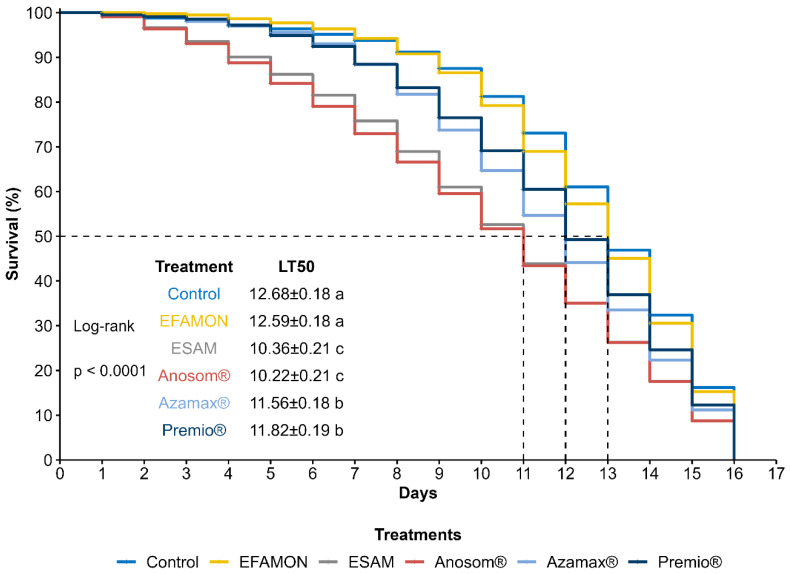
Survival curves and lethal time (LT_50_) (±SE) of the F_0_ generation of *Trichogramma atopovirilia* exposed to *Ephestia kuehniella* eggs contaminated with botanical and synthetic insecticides. Lethal times followed by different letters are significantly different (pairwise comparisons using log-rank test, *p* < 0.05). EFAMON (aqueous emulsion of the methanolic fraction of the ethanolic leaf extract of *Annona montana*); ESAM (aqueous emulsion of the ethanolic seed extract of *Annona mucosa*). Temperature: 25 ± 1 °C, 60 ± 10% UR and 14 h photophase.

Survival of the F_0_ generation of *T.atopovirilia* was significantly affected by the treatments (log-rank test: χ^2^ = 103.60; d.f. = 5, *p* < 0.001). The botanical insecticides ESAM and Anosom^®^ resulted in faster mortality rates, with median lethal times (LT_50_) of 10.36 days and 10.22 days, respectively. The control treatment exhibited an LT_50_ of 12.68 days, which was not significantly different from EFAMON (LT_50_ = 12.59 days). In contrast, Azamax^®^ and Premio^®^ had similar median lethal times of 11.56 and 11.82 days, respectively (Figure 2).

**Figure 3:**
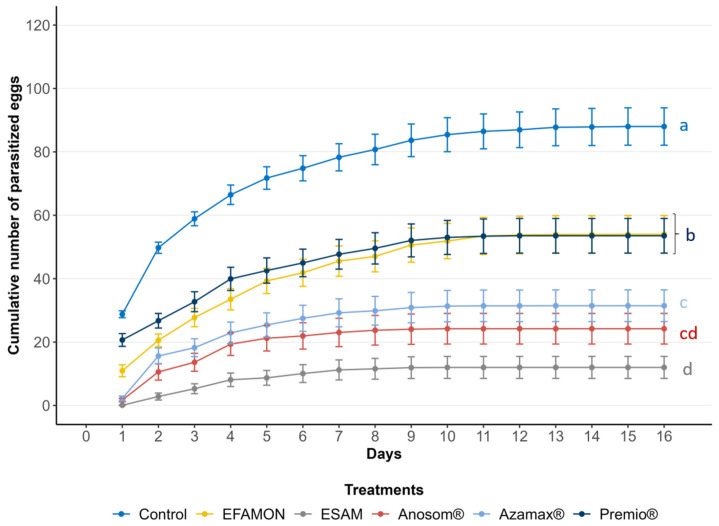
Cumulative parasitism (mean number of eggs, ±SE) of the F_0_ generation of *Trichogramma atopovirilia* exposed to *Ephestia kuehniella* eggs contaminated with botanical and synthetic insecticides. Curves with different lowercase letters differ significantly among themselves (Tukey, *p* < 0.05). EFAMON (aqueous emulsion of the methanolic fraction of the ethanolic leaf extract of *Annona montana*); ESAM (aqueous emulsion of the ethanolic seed extract of *Annona mucosa*). Temperature: 25 ± 1 °C, 60 ± 10% UR and 14 h photophase.

The insecticides significantly affected parasitism (F_5,170_ = 74.962; *p* < 0.001). Females from the F_0_ generation that were exposed to the ESAM parasitized less than 1% of the eggs within 24 h. This resulted in a 99.76% reduction in parasitism compared to the control group, leading to the classification of this treatment as toxic (class 4). The bioinsecticides based on acetogenins (Anosom^®^) and limonoids (Azamax^®^) demonstrated reductions in parasitism of 99.13% and 92.36%, respectively, and were classified as moderately toxic (class 3). In contrast, females exposed to the EFAMON treatment parasitized 7.28% of the eggs, which was statistically different from the control group, resulting in a 62% reduction in parasitism and a classification of slightly toxic (class 2). The synthetic insecticide Premio^®^ did not show any significant difference from the negative control in terms of parasitism and was considered innocuous (Table 2). Figure 3 illustrates the accumulated parasitism throughout the lifespan of the females (F_5,169_ = 22.894; *p* < 0.001). At the end of the females’ life cycle, no statistical differences in parasitism were observed between the EFAMON and Premio^®^ treatments, or between Azamax^®^ and Anosom^®^. However, ESAM resulted in the lowest number of parasitized eggs overall.

### 3.2. Transgenerational Effects on the F_1_ and F_2_ Generations

No adults emerged from eggs parasitized by parents that had contact with the botanical insecticide ESAM. Due to the low parasitism observed in ESAM, the evaluation of other parameters was not conducted for this treatment. Conversely, EFAMON, Azamax^®^, and Premio^®^ resulted in emergence rates above 70% and did not differ statistically from the negative control (82%). The botanical insecticide Anosom^®^ showed an emergence rate of 61.40%, representing a 25.17% reduction compared to the control (χ^2^ = 10.17; d.f. = 4; *p* = 0.038). Thus, all treatments affected the emergence parameter of the F_1_ generation but were classified as harmless. No effects of the treatments were observed on the sex ratio of this generation (F_4,86_ = 1.045; *p* = 0.3888) (Table 3).

**Figure 4:**
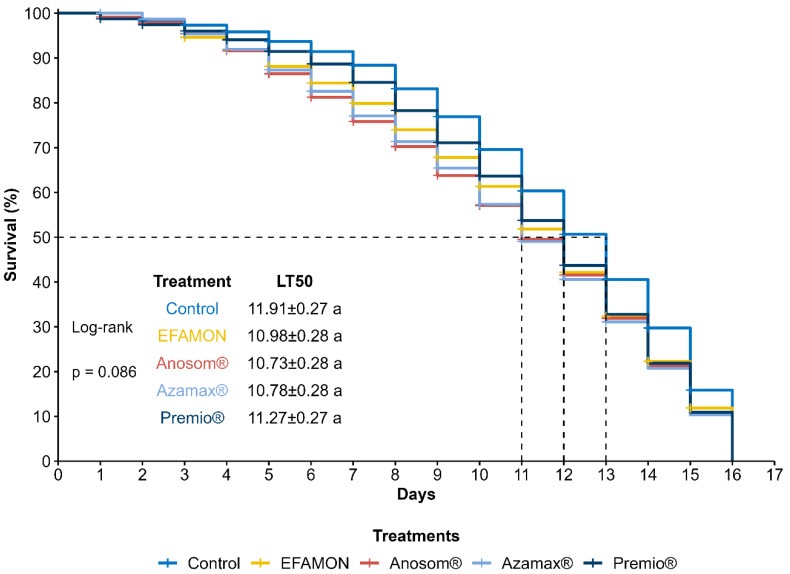
Survival curves and lethal time (LT_50_) (±SE) of the F_1_ generation of *Trichogramma atopovirilia* exposed to *Ephestia kuehniella* eggs contaminated with botanical and synthetic insecticides. Lethal times followed by different letters are significantly different (pairwise comparisons using log-rank test, *p* < 0.05). EFAMON (aqueous emulsion of the methanolic fraction of the ethanolic leaf extract of *Annona montana*); ESAM (aqueous emulsion of the ethanolic seed extract of *Annona mucosa*). Temperature: 25 ± 1°C, 60 ± 10% UR and 14 h photophase.

**Figure 5:**
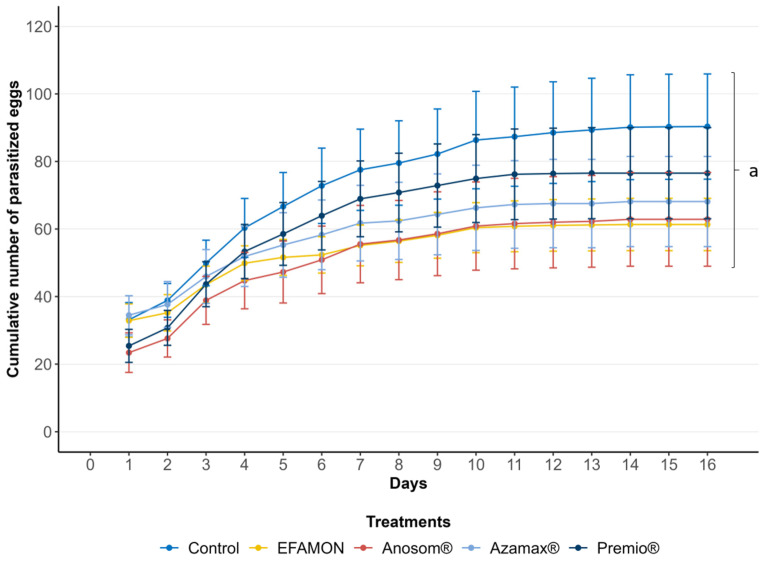
Cumulative parasitism (mean number of eggs, ± SE) of the F_1_ generation of *Trichogramma atopovirilia* exposed to *Ephestia kuehniella* eggs contaminated with botanical and synthetic insecticides. Curves followed by different letters are significantly different (Tukey, *p* < 0.05). EFAMON (aqueous emulsion of the methanolic fraction of the ethanolic leaf extract of *Annona montana*); ESAM (aqueous emulsion of the ethanolic seed extract of *Annona mucosa*). Temperature: 25 ± 1 °C, 60 ± 10% UR and 14 h photophase.

The survival of the F_1_ generation was not affected by the treatments (log-rank test: χ^2^ = 8.15; d.f. = 4, *p* = 0.086). There were no significant differences in lethal times (LT_50_), which were approximately 11 days (Figure 4). Additionally, parasitism rates in the F_1_ generation remained unchanged, both within the first 24 h (F_4,70_ = 0.8992; *p* = 0.4693) and over the accumulated time (F_4,70_ = 0.8192; *p* = 0.5172). All insecticides tested were classified as harmless, with each causing less than a 30% reduction in parasitism compared to the control group (Table 4 and Figure 5). The same trend was observed in the F_2_ generation, with no significant effects on the emergence rates (F_4,51_ = 0.7298; *p* = 0.5758) or sex ratio (F_4,48_ = 0.6570; *p* = 0.6248) (Table 5).

**Figure 6:**
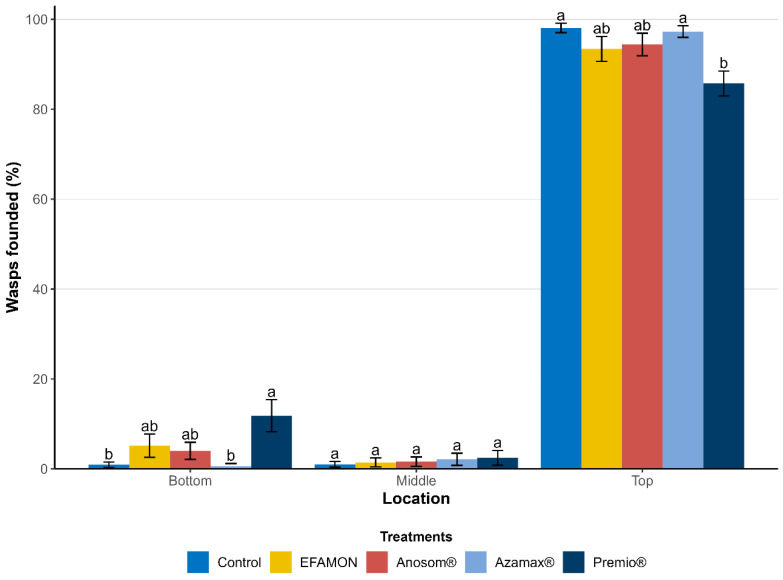
Flight capacity of the F_2_ generation of *Trichogramma atopovirilia* exposed to *Ephestia kuehniella* eggs contaminated with botanical and synthetic insecticides. Percentages of females found in each location within the flight cage followed by different letters are significantly different (Tukey test, *p* < 0.05). EFAMON (aqueous emulsion of the methanolic fraction of the ethanolic leaf extract of *Annona montana*); ESAM (aqueous emulsion of the ethanolic seed extract of *Annona mucosa*). Temperature: 25 ± 1 °C, 60 ± 10% UR and 14 h photophase.

Most of the F_2_ generation adults successfully reached the top of the cage and were classified as fliers. However, significant differences were observed among the treatments (F_8,75_ = 5.8785; *p* < 0.001). The insecticide Premio^®^ resulted in the lowest percentage of flying adults compared to the control group. Nevertheless, over 80% of the treated individuals were still capable of flight (Figure 6).

## 4. Discussion

To improve compatibility between chemical insecticides and biological control agents of *S. frugiperda*, this study investigates the lethal and transgenerational effects of botanical and synthetic insecticides on the egg parasitoid *T. atopovirilia*, enhancing understanding of insecticide selectivity for IPM programs. Adults of *Trichogramma* spp. reach their peak parasitism within the first days of life [12,43]. Our results indicated that the median lethal time (LT_50_) for the exposed individuals ranged from 10 to 12 days, providing enough time for parasitism to occur. However, we observed a negative effect on this parameter. F_0_ females exposed to the botanical insecticide ESAM (aqueous emulsion of the ethanolic seed extract of *Annona mucosa*) parasitized less than 1% of the eggs within 24 h, categorizing it as toxic (class 4). Due to the drastic reduction in parasitism, no emergence was observed, and it was not possible to evaluate this treatment in subsequent bioassays. While ESAM has shown effectiveness in controlling various agricultural pests, this study is the first to report its selectivity evaluation on *T. atopovirilia*. In a related study, Bernardi et al. [44] demonstrated that this extract was toxic to another group of parasitoids, *Trichopria anastrephae* Lima (Hymenoptera: Diapriidae), resulting in 70% mortality by contact.

The ESAM extract is primarily composed of acetogenins, particularly rolliniastatin-1 [45]. *Trichogramma atopovirilia* females might have been exposed to these compounds through tarsal contact or their ovipositor during parasitism, leading to intoxication. Acetogenins have various modes of action, including the inhibition of complex I in the mitochondrial electron transport chain, which compromises ATP production [46,47]. Additionally, there are reports of effects on the digestive system and interactions with membrane cell phosphates [18,19,48,49]. Another possible reason for the observed lack of parasitism is repellency. Other compounds present in the ESAM extract, alongside the acetogenins, even in minor quantities, could also have a repellent effect [50,51,52]. Baldin et al. [53] demonstrated that ESAM has a repellent effect on *Aphis glycines* Matsumura (Hemiptera: Aphididae). Although these insects belong to a different group, it is plausible that the parasitoids studied here were similarly affected.

Anosom^®^ and EFAMON (aqueous emulsion of the methanolic fraction of the ethanolic leaf extract of *Annona montana*) reduced parasitism in the F_0_ generation. Anosom^®^ is classified as moderately toxic (class 3), while EFAMON is considered slightly toxic (class 2). Both products are derived from plants of the same genus as ESAM, *Annona*, and predominantly contain acetogenins as active compounds [54]. However, their impact on parasitism was less pronounced than that of ESAM. These differences can be attributed to variations in the chemical structures of the acetogenins present in each product. Anosom^®^ is primarily based on the acetogenin annonin, while EFAMON includes a variety of acetogenins (montanacin-L, montanacin-K, montanacin-D, and montanacin-E) [16,17]. The biological activity of each compound depends directly on its chemical structure; factors such as alkyl chain length, ring presence, functional group quantity, and degree of unsaturation can significantly affect biological performance [45,52,54,55].

The commercial botanical insecticide Azamax^®^, based on limonoids (azadirachtin + 3-tigloilazadirachtol), reduced parasitism in the F_0_ generation by 92% (class 3 = moderately toxic) compared to the control. These results are consistent with those reported by Rodrigues et al. [56], who observed a 79.73% reduction in the parasitism of *T. atopovirilia* when exposed to eggs contaminated with the same insecticide. Similarly, Luckman et al. [57] observed comparable effects on parasitism reduction in *T. pretiosum*. Azadirachtin affects insects through various mechanisms, including antifeedant, repellent, and ovicidal effects [58,59]. One possible reason for the reduced parasitism observed in this study is sterility. Previous studies have documented hormonal changes that adversely affect egg viability in insects exposed to azadirachtin [60]. Therefore, it is likely that the affected insects’ reproductive capacity was compromised, resulting in the laying of non-viable eggs. Another factor to consider is the repellent effect of azadirachtin, as it belongs to the limonoid group, compounds well known for this characteristic [61,62].

The synthetic insecticide Premio^®^ (chlorantraniliprole) did not affect F_0_ generation parasitism in the first 24 h (class 1 = innocuous). Its mode of action involves the activation of ryanodine receptors, leading to the continuous release of calcium ions from the sarcoplasmic reticulum into the cytoplasm of insect muscle cells [63,64]. A possible explanation for the absence of effects observed is that, although chlorantraniliprole acts through contact and ingestion, its effect is more pronounced when ingestion occurs, a pathway to which females were not exposed in this study [64]. Our findings align with those of Santos et al. [65], who reported that chlorantraniliprole was selective for *T. pretiosum*, resulting in less than a 30% reduction in parasitism. This is also consistent with the study conducted by Li et al. [66], who demonstrated the selectivity of chlorantraniliprole for several *Trichogramma* species, including *T. dendrolim* Matsumura, *T. chilonis* Ishii, and *T. mwanzai* Schulten & Feijen (Hymenoptera: Trichogrammatidae). When considering the full lifespan of the females, Premio^®^ resulted in a significant reduction in parasitism compared to the control. This effect may be attributed to its prolonged mode of action, as its primary impact on muscle function likely takes longer to manifest [63,64]. Despite this, the reduction in parasitism did not exceed 35%.

We investigated the transgenerational effects of tested insecticides, as these compounds can alter not only the individuals that are directly exposed but also subsequent generations, resulting in long-term impacts [67]. Our objective was to determine whether parents exposed to insecticides could negatively affect both their offspring (F_1_) and their descendants (F_2_), who had no direct contact with these compounds. Transgenerational effects may influence survival, parasitism, sex ratio, and even behavior [33,34]. However, our results indicated no transgenerational effects on survival, parasitism, or sex ratio in the F_1_ generation, nor on emergence and sex ratio in the F_2_ generation. Interestingly, even botanical insecticides, which are classified as toxic or moderately toxic in the parental generation, were found to be innocuous to subsequent generations. These findings contrast with results reported for synthetic insecticides, as demonstrated by Silva et al. [68] and Cantori et al. [22], who observed transgenerational effects on *T. atopovirilia*, such as changes in longevity, parasitism, and sex ratio. Premio^®^ significantly reduced flight capacity compared to the control group. Although this insecticide did not cause direct mortality in the parental generation, exposure to Premio^®^ may have induced physiological changes that affected wing development. These sublethal effects could have been inherited, resulting in impaired flight capacity in the F_2_ generation [69,70].

## 5. Conclusions

Our results emphasize the importance of evaluating insecticide selectivity, including transgenerational effects. Even products derived from the same botanical family exhibited varying effects on *T. atopovirilia*. Most botanical insecticides, except for ESAM, had adverse effects on the parental generations but were harmless to subsequent generations. These findings suggest that such insecticides could be strategically utilized during pest outbreaks in integrated pest management (IPM) programs, minimizing disruptions to biological control methods. Further research, particularly under semi-field and field conditions, is necessary for a deeper understanding. Nonetheless, this study significantly contributes to the potential integration of chemical and biological control strategies for managing *S. frugiperda* in an environmentally sustainable way.

## Figures and Tables

**Table 1:** Lethal concentrations (LC_90_) ^1^ of the treatments estimated for *Spodoptera frugiperda* and used in selectivity bioassays on *Trichogramma atopovirilia*.

Treatments	*N*	LC_90_ (95% CI) ^1^	Slope ± SE	*X* ^2^ *(df)*	*p*	Manufacturer
EFAMON	452	10,949.0 mg kg^−1^ (8115.5–14,771.8)	1.93 ± 0.20	9.39 (5)	0.094	Pre-commercial
ESAM	672	1882.0 mg kg^−1^ (13,486.0–3830.0) ^2^	3.44 ± 0.64	9.24 (4)	0.838	Pre-commercial
Anosom^®^ 1 EC, 1 g a.i L^−1^ (Acetogenin annonin)	480	2959.0 mg kg^−1^ (2596.0–3504.0) ^3^	3.53 ± 0.41	0.78 (2)	0.160	Agri Life (Hyderabad, India)
Azamax^®^ 1.2 EC, 1.2 g a.i L^−1^ (Azadirachtin + 3-tigloylazadirachtol)	442	65.42 mg kg^−1^ (44.28–96.68)	1.62 ± 0.16	8.04 (4)	0.090	UPL Brazil (Campinas, Brazil)
Premio^®^ SC, 200 g a.i. L^−1^ (Chlorantraniliprole)	523	0.43 mg kg^−1^ (0.37–0.50)	3.28 ± 0.28	2.58 (4)	0.630	FMC Brazil (Campinas, Brazil)

EFAMON = Aqueous emulsion of the methanolic fraction of the ethanolic extract of *Annona montana* leaves. ESAM = Aqueous emulsion of the ethanolic extract of *Annona mucosa* seeds. ^1^ LC_90_ = Lethal concentration and confidence interval (95%) required to kill 90% of first-instar larvae of *S. frugiperda* after seven days of exposure. Concentrations obtained from ^2^ Ansante et al. [15] and ^3^ Ansante et al. [16].

**Table 2:** Parasitism of the F_0_ generation of *Trichogramma atopovirilia* after exposure to *Ephestia kuehniella* eggs contaminated with botanical and synthetic insecticides.

F_0_			
Treatments	24 h Parasitism (%) ^1^	Parasitism Reduction (%)	IOBC/WPRS Classification ^2^
Control	19.19 ± 0.72 a		
EFAMON	7.28 ± 1.25 b	62.02	2
ESAM	0.04 ± 0.05 d	99.76	4
Anosom^®^	1.12 ± 0.36 cd	94.13	3
Azamax^®^	1.46 ± 0.52 c	92.36	3
Premio^®^	13.77 ± 1.34 a	28.21	1

^1^ Means (±SE) followed by different letters are significantly different (Tukey test, *p* < 0.05). ^2^ IOBC/WPRS classifications: class 1 = innocuous (<30% reduction), class 2 = slightly toxic (30% to 80% reduction), class 3 = moderately toxic (>80% to 99% reduction), class 4 = toxic (>99% reduction). Temperature: 25 ± 1 °C, 60 ± 10% UR and 14 h photophase.

**Table 3:** Emergence of the F_1_ generation of *Trichogramma atopovirilia* after exposure to *Ephestia kuehniella* eggs contaminated with botanical and synthetic insecticides.

F_1_				
Treatments	24 h Emergence (%) ^1^	Emergence Reduction (%)	IOBC/WPRS Classification ^2^	Sex Ratio
Control	83.23 ± 2.64 ab			0.64 ± 0.03
EFAMON	87.24 ± 3.45 a	9.30	1	0.65 ± 0.07
ESAM	-	-	-	-
Anosom^®^	61.39 ± 11.08 b	25.17	1	0.77 ± 0.12
Azamax^®^	70.89 ± 10.93 ab	13.59	1	0.69 ± 0.26
Premio^®^	85.26 ± 2.33 ab	0.00	1	0.54 ± 0.12

^1^ Means (±SE) followed by different letters are significantly different (Tukey test, *p* < 0.05). ^2^ IOBC/WPRS classifications: class 1 = innocuous (<30% reduction), class 2 = slightly toxic (30% to 80% reduction), class 3 = moderately toxic (>80% to 99% reduction), class 4 = toxic (>99% reduction). Temperature: 25 ± 1 °C, 60 ± 10% UR and 14 h photophase.

**Table 4:** Parasitism of the F_1_ generation of *Trichogramma atopovirilia* after exposure to *Ephestia kuehniella* eggs contaminated with botanical and synthetic insecticides.

F1			
Treatments	24 h Parasitism (%) ^1^	Parasitism Reduction (%)	IOBC/WPRS Classification ^2^
Control	22.08 ± 3.39 a	-	-
EFAMON	21.91 ± 3.29 a	0.80	1
Anosom^®^	22.57 ± 3.34 a	29.38	1
Azamax^®^	22.97 ± 2.30 a	0.00	1
Premio^®^	16.93 ± 3.26 a	23.34	1

^1^ Means (± SE) followed by different letters do not statistically differ from each other (Tukey test, *p* < 0.05). ^2^ IOBC/WPRS classifications: class 1 = innocuous (<30% reduction), class 2 = slightly toxic (30% to 80% reduction), class 3 = moderately toxic (>80% to 99% reduction), class 4 = toxic (>99% reduction). Temperature: 25 ± 1 °C, 60 ± 10% UR and 14 h photophase.

**Table 5:** Emergence of the F_2_ generation of *Trichogramma atopovirilia* after exposure to *Ephestia kuehniella* eggs contaminated with botanical and synthetic insecticides.

F_2_				
Treatments	24 h Emergence (%) ^1^	Emergence Reduction (%)	IOBC/WPRS Classification ^2^	Sex Ratio
Control	83.55 ± 4.06 a	-	-	0.62 ± 0.06
EFAMON	73.65 ± 5.33 a	11.86	1	0.69 ± 0.04
ESAM	-	-	-	-
Anosom^®^	69.09 ± 8.16 a	17.31	1	0.60 ± 0.16
Azamax^®^	81.37 ± 3.82 a	2.62	1	0.82 ± 0.13
Premio^®^	79.20 ± 4.74 a	5.22	1	0.81 ± 0.19

^1^ Means (±SE) followed by different letters are significantly different (Tukey test, *p* < 0.05). ^2^ IOBC/WPRS classifications: class 1 = innocuous (<30% reduction), class 2 = slightly toxic (30% to 80% reduction), class 3 = moderately toxic (>80% to 99% reduction), class 4 = toxic (>99% reduction). Temperature: 25 ± 1 °C, 60 ± 10% UR and 14 h photophase.

## Data Availability

The original contributions presented in this study are included in the article/Supplementary Material. Further inquiries can be directed to the corresponding author.

## Supplementary Materials

The following supporting information can be downloaded at: https://www.mdpi.com/article/10.3390/insects16050493/s1, Supplementary Material S1. The document provides a detailed description of the procedure used to determine the lethal concentrations (LC_90_) for *Spodoptera frugiperda*, which were subsequently used in selectivity bioassays with *Trichogramma atopovirilia*. It includes step-by-step methodologies for preparing the pre-formulations and the process for generating concentration-mortality curves.
