# Supplementary material for: Harmful to Parents, Harmless to Offspring: Lethal and Transgenerational Effects of Botanical and Synthetic Insecticides on the Egg Parasitoid Trichogramma atopovirilia
Source: Insects. 2025 May 5;16(5):493. doi: 10.3390/insects16050493 (PMC12112051; doi:10.3390/insects16050493)
Supplement: Supplementary file 1 [file insects-16-00493-s001.zip › insects-3520518-supplementary.pdf]

Supplementary material (S1)
https://doi.org/10.5281/zenodo.14913959

Harmful to Parents, Harmless to Offspring: Lethal and Transgenerational Effects of Botanical and Synthetic Insecticides on the Egg Parasitoid *Trichogramma atopovirilia*

This document outlines the process for determining the lethal concentrations (LC_{90}) used in selectivity bioassays with *Trichogramma atopovirilia.* Initially, the procedure for preparing the pre-formulations is presented, followed by a description of the development of the concentration-mortality curves.

Preparation of botanical pre-formulations from A. montana and A. mucosa

Ethanolic extracts from the leaves of *A. montana* and the seeds of *A. mucosa* were used to develop botanical pre-formulations. The leaves and seeds were first dried in an oven at 38°C for 48 hours, then ground using a knife mill, and subjected to ethanol extraction. After a three-day resting period, the extract was filtered, and the solvent was removed using a rotary evaporator set at 50°C and -600 mmHg

For *A. montana*, a liquid-liquid partitioning was performed to separate the phases with different chemical affinities, resulting in the methanolic fraction. To prepare emulsions from the methanolic fraction of *A. montana* and the ethanolic extract of *A. mucosa*, surfactants Triton® X-100 (0.1%, v/v) and Tween® 80 (1%, v/v) were added, along with a mixture of acetone and methanol (1%, v/v) in a 1:1 (v/v) ratio. These components were used to reduce the water's surface tension, improve leaf adhesion, and facilitate emulsion dilution.

Determination of concentration-mortality curves

Concentration-mortality curves were conducted to determine the lethal concentration required for the treatments to cause mortality in the pest *S. frugiperda*. At least eight different concentrations were tested and incorporated into 100g of artificial diet. After preparation, 1 mL of the contaminated diet was distributed into the wells of 128-cell plastic plates (Advento do Brasil, São Paulo, Brazil) using a hypodermic syringe (BD PlastipaK™). The plates were infested with neonate larvae (less than 24 hours old) and sealed with transparent plastic lids to allow aeration. Mortality was monitored daily until seven days.

For the EFAMON treatment, the concentrations were pre-diluted in 6 mL of a solvent solution comprising acetone and methanol in a 1:1 ratio before being added to the diet. The same solvents were used for the control treatment. The experiment was designed using a completely randomized approach, with four replicates of 16 insects per concentration in each treatment. Regarding ESAM and Anosom®, their concentration-response curves had already been determined in previous studies [15,16], following the same protocol outlined in this document.
